# How to modulate the formation of negative volatile sulfur compounds during wine fermentation?

**DOI:** 10.1093/femsyr/foab038

**Published:** 2021-06-30

**Authors:** Rafael Jimenez-Lorenzo, Audrey Bloem, Vincent Farines, Jean-Marie Sablayrolles, Carole Camarasa

**Affiliations:** SPO, Univ Montpellier, INRAE, Institut Agro, Montpellier, France; SPO, Univ Montpellier, INRAE, Institut Agro, Montpellier, France; SPO, Univ Montpellier, INRAE, Institut Agro, Montpellier, France; SPO, Univ Montpellier, INRAE, Institut Agro, Montpellier, France; SPO, Univ Montpellier, INRAE, Institut Agro, Montpellier, France

**Keywords:** yeast metabolism, wine fermentation, nutrient availability, *S. cerevisiae*, diversity, volatile sulfur compounds

## Abstract

Beyond the production of positive aromas during alcoholic fermentation, *Saccharomyces cerevisiae* metabolism also results in the formation of volatile compounds detrimental to wine quality, including a wide range of volatile sulfur compounds (VSCs). The formation of these VSCs during wine fermentation is strongly variable and depends on biological and environmental factors. First, the comparison of the VSCs profile of 22 *S. cerevisiae* strains provided a comprehensive overview of the intra-species diversity in VSCs production: according to their genetic background, strains synthetized from 1 to 6 different sulfur molecules, in a 1- to 30-fold concentration range. The impact of fermentation parameters on VSCs production was then investigated. We identified yeast assimilable nitrogen, cysteine, methionine and pantothenic acid contents – but not SO_2_ content – as the main factors modulating VSCs production. In particular, ethylthioacetate and all the VSCs deriving from methionine catabolism displayed a maximal production at yeast assimilable nitrogen concentrations around 250 mg/L; pantothenic acid had a positive impact on compounds deriving from methionine catabolism through the Ehrlich pathway but a negative one on the production of thioesters. Overall, these results highlight those factors to be taken into account to modulate the formation of negative VSCs and limit their content in wines.

## Teaser

The influence of both *Saccharomyces cerevisiae* strains and environmental factors on the formation of volatile sulfur compounds causing off-flavors during wine fermentation was investigated.

## INTRODUCTION

Wine aroma is one of the most important determinants of quality, because the perception of volatile molecules through their interactions with the consumer's olfactory senses strongly contributes to the acceptance or rejection of the product. Wine aroma consists of thousands of volatile molecules from different origins: the primary or varietal aromas, present in berries and typical of the grape variety; the secondary aromas produced through yeast metabolism during alcoholic fermentation and, finally, the tertiary or post-fermentative aromas, due to chemical reactions during ageing (Robinson *et al*. 2014).

In wines, volatile sulfur compounds (VSCs) are an important class due to their abundance and their strong impact on the final quality of wines due to a very low perception threshold. Sulfur compounds are considered as a ‘double edged-sword’ as some of them positively contribute to wine aroma, whereas others are considered as off-flavors (reductive aromas) undesirable in wines. Indeed, the implementation of strategies promoting the formation of positive VSCs is often accompanied by an increase in the formation of sulfur off-flavors (Lattey *et al*. 2007), highlighting the importance to comprehensively address the formation of sulfur-containing molecules during winemaking.

Generally, mercaptans with a positive impact on wine quality, such as 3-mercaptohexan-1-ol (3MH), 3-mercaptohexyl acetate (3MHA) or 4-mercapto-4-methylpentan-2-one (4MMP) originate from precursors in grapes, mainly as glutathionylated, cysteinylated, cysteinyl-glycine or glutamyl-cysteine S- conjugated forms (Tominaga *et al*. 1998; Bonnaffoux *et al*. 2021). Once the thioester bond in precursors is cleaved through β-lyase and/or endopeptidase activities, these varietal aromas provide wines with notes of box tree, grapefruit or passion fruit (Tominaga *et al*. 2000). By contrast, a majority of VSCs originating from yeast metabolism during alcoholic fermentation have a detrimental effect on wine quality. The profile of VSCs produced by yeast during fermentation is highly dependent on the yeast strain/species used (Spinnler *et al*. 2001, Moreira *et al*. 2008; Patrignani *et al*. 2016). Usually associated to rotten egg, cabbage, garlic, onion, cheese or burnt rubber notes, VSCs are often referred to as reduced or reductive aromas. They include hydrogen sulfide (H_2_S), methanethiol (MeSH), ethanethiol (EtSH), dimethyl sulfide (DMS), diethyl sulfide (DES), dimethyl disulfide (DMDS), ethylthio acetate (ETA), S-methylthio acetate (SMTA) or methionol (ME) among others (Smith *et al*. 2015). VSCs separate in highly volatile compounds characterized by a boiling point lower than 90°C, which can be easily removed from wine through simple racking or aeration, or volatile compounds with boiling points over 90°C, more difficult to eliminate through evaporation (Moreira *et al*. 2002).

Due to its very low sensory perception threshold, the metabolism of H_2_S by *Saccharomyces cerevisiae* during wine fermentation has been extensively characterized. Briefly, this compound is formed from inorganic sulfur compounds (sulphate and possibly sulfite) through the sulphate assimilatory pathway (Cherest and Surdin-Kerjan 1992; Thomas and Surdin-Kerjan 1997) and can further combine with O-acetyl homoserine as the first step in the biosynthesis of the sulfur amino acids cysteine and methionine (Spiropoulos *et al*. 2000; Swiegers and Pretorius 2007; Landaud, Helinck and Bonnarme 2008). The different genes involved in the biosynthesis of these sulfur-containing amino acids as well as the underlying molecular mechanisms have been well-described (Huang *et al*. 2017; Noble, Sanchez and Blondin 2015; Ugliano *et al*. 2009; Linderholm *et al*. 2008; Spiropoulos *et al*. 2000). Conversely, information on metabolic precursors and pathways involved in the formation of other VSCs is incomplete, and mainly concerns the formation of ME, 3-(methylthio)-propanoic acid (3MTPA), (methylthio)-propyl acetate (3MTPAc) and ethyl 3-(methylthio)-propanoic acid (E3MTP), which derive, through the Ehrlich pathway, from keto- (methylthio) butyric acid, a common intermediate with the methionine biosynthesis pathway. It has also been suggested that the catabolism of cysteine and homocysteine through the Ehrlich pathway may respectively result in the production of 2-mercaptoethanol (2ME) and 4-methylthiobutanol (4MTB; Moreira *et al*. 2002; Mestres, Busto and Guasch 2000; Swiegers *et al*. 2005). As regards highly volatile thiols, an S-lyase enzymatic activity and/or a non-enzymatic process may contribute to the formation of MeSH (Perpète *et al*. 2006), while ethanethiol likely originates from a chemical or biological combination of H_2_S with ethanol or acetaldehyde (Rauhut 2017). Other compounds, such as 2-Methylthioethanol (2MTE), ethylthiopropanol (ETP) or 2-methyl-tetrahydrothiophen-3-one (MTHTP) were also detected in wines with reductive off-odors, but their origin remains to be elucidated. Finally, it is important to also consider that VSCs are extremely active chemically, and can evolve depending on medium physicochemical parameters or react with one another or with other compounds resulting in the formation of additional molecules (Vela *et al*. 2018; Kinzurik *et al*. 2020). As an example, dimerization of ethanethiol by creation of a disulfur bond results in the formation of diethyl disulfide (DEDS).

As a consequence, VSC concentrations in wines depend on both environmental factors (grape juice content in nutrient and heavy metals) and on the technological itineraries set up for vine cultivation and winemaking, as phytosanitary treatment, addition of SO_2_, yeast species/strains carrying out the fermentation, use of clarification… In particular, H_2_S formation during fermentation is modulated by yeast assimilable nitrogen (YAN), pantothenic acid and biotin availability (Bohlscheid *et al*. 2011; Ugliano *et al*. 2008). Thus, while, in presence of nitrogen, excess inorganic sulfur was used to a large extent for the synthesis of sulfur amino acids from O-acetylhomoserine, insufficient availability of this precursor when nitrogen is limiting resulted in sulphate accumulation within cells and later diffusion into the medium. Then, the reductive conditions related to low pH and anaerobiosis favored the spontaneous conversion of sulphate into H_2_S (Jiranek, Langridge and Henschke 1996; Swiegers and Pretorius 2007; Rauhut 2017). In the same way, low concentrations of pantothenic acid, a vitamin (B5) used as cofactor in coenzyme A synthesis (Sandoval *et al*. 2014), increased H_2_S production during wine fermentation, an increase likely related to a deficiency in O-acetylhomoserine (Edwards and Bohlscheid 2007). It has been additionally reported that H_2_S formation was enhanced when cysteine was provided in the medium in large excess, i.e. over 50 mg/L (Robinson *et al*. 2014). Methionine and cysteine are amino acids in low concentrations in grape juice, normally below 10 mg/L (Petrovic, Aleixandre-Tudo and Buica 2019). However, taking into account their catabolic network, these sulfur amino acids can be considered as important precursors for VSCs synthesis. Their effective contribution, and more generally, the impact of environmental factors on the formation of unpleasant sulfur compounds, until now poorly documented, is worthy of investigation.

In this study, we evaluated the variability of VSCs formation during wine fermentation, first investigating intra-species diversity in *S. cerevisiae* strains regarding their capacity to produce these molecules. Then, we examined the incidence of environmental parameters identified as the most relevant to modulate VSCs production, i.e. YAN, pantothenic acid and sulfur amino acids availability.

## MATERIAL AND METHODS

### Yeast strains

In this study, 22 *S. cerevisiae* strains were selected (Table 1). Eleven were provided by Lallemand SA (Montreal, Canada); the rest (11) were selected from the INRAE yeast collection in which strains are stored at −80°C in a mix of YEPD medium (10 g/L yeast extract, 20 g/L peptone and 20 g/L dextrose) and glycerol (15%). Cryopreserved yeast cultures were thawed at room temperature and streaked on Yeast Peptone Dextrose (YPD) agar. For precultures, one colony of each strain was used to inoculate a sterile 50 mL tube containing 20 mL YEPD medium. Strains were grown for 24 h at 28°C with a 190 rpm agitation. After that, tubes were centrifuged during 5 min (4500 rpm, 4°C) and the pellet was washed twice with MilliQ® H_2_O before resuspension in 15 mL of water containing 9 g/L NaCl. Finally, 1 × 10^6^ CFU/mL were inoculated in each fermentor to carry out fermentation. All fermentations were performed in duplicate.

**Table 1.** *S. cerevisiae* yeast strains used in this study.

Name	Origin	Genotype
IR01	Lallemand	Mat a/ Mat α
IR02	Lallemand	Mat a/ Mat α
IR03	Lallemand	Mat a/ Mat α
ECA5	Lallemand	Mat a/ Mat α
UCD522	INRAe	Mat a/ Mat α
IR4	Lallemand	Mat a/ Mat α
IR5	Lallemand	Mat a/ Mat α
EC1118	INRAe	Mat a/ Mat α
IR06	Lallemand	Mat a/ Mat α
IR07	Lallemand	Mat a/ Mat α
IR08	Lallemand	Mat a/ Mat α
L85	Lallemand	Mat a/ Mat α
MTF 1615	INRAe	Mat a/ Mat α
MTF 1764	INRAe	Mat a/ Mat α
K1	INRAe	Mat a/ Mat α
MTF2414	INRAe	Mat a/ Mat α
MTF 2292	INRAe	Mat a/ Mat α
IR10	INRAe	Mat a/ Mat α
MTF 914	INRAe	Mat a/ Mat α
IR11	INRAe	Mat a/ Mat α
MTF 2113	INRAe	Mat a/ Mat α
MTF1438	INRAe	Mat a

### Fermentation media

A synthetic must (SM), as described by Rollero *et al*. 2015, that mimics normal grape must conditions was used in this study. SM nitrogen was composed of ammonium chloride and amino acids. The stock solution of amino acids was composed of (in milligrams per liter): proline (288.28), alanine (68.37), arginine (176.17), aspartic acid (20.94), L-Cysteine (6.16), glutamine (237.77), glutamic acid (56.67), glycine (8.624), histidine (15.4), isoleucine (15.4), leucine (22.79), lysine (8.008), methionine (14.78), phenylalanine (17.86), serine (36.96), threonine (35.73), tryptophan (84.39), tyrosine (8.62) and valine (20.94. A concentration of 2 mg/L β-phytosterols was added to the SM to satisfy yeast requirements during anaerobic growth. The stock solution was composed of 2 g/L phytosterols in a mix of Tween 80 and absolute ethanol (1:1, v/v).

#### Nitrogen impact

To test the impact of nitrogen on VSCs production, four different conditions were assayed with 100, 200, 300 and 400 mg/L YAN. To achieve this, SM was supplemented with a mix of an amino acids solution with NH_4_Cl.

#### Pantothenic acid impact

We added different concentrations of pantothenic acid (10, 25, 50, 100 and 250 µg/L) to SM with 200 mg/L YAN (devoid of pantothenic acid) to find out the impact of this vitamin on VSCs production.

#### Sulfur amino acids impact

To assay the impact that sulfur amino acids may have on the modulation of VSCs production, we increased 20-fold the concentrations of either methionine or cysteine and of methionine plus cysteine. Conversely, we also tested media depleted of methionine, cysteine, or methionine plus cysteine. All these media, summarized in Table 2, had similar assimilable nitrogen with slight variations only in the ratio between amino acids and ammonium nitrogen.

**Table 2.** Composition of the nitrogen source of different media used to evaluate the impact of sulfur amino acids in VSCs production. YAN concentration was 200 mg N/L regardless of the conditions.

Condition	20 × Met	20 × Cys	20 × Met/Cys	O Met	0 Cys	0 Met/Cys
Methionine (mg/L)	295.7	14.8	295.7	0	14.8	0
Cysteine (mg/L)	6.2	123.2	123.2	6.2	0	0
Amino acids solution (mL)	6.2	6.2	6.2	6.2	6.2	6.2
NH4Cl (mg/L)	190.3	201.9	187.1	217.6	216.89	218.4

Fermentations were carried out at 22°C in 300 mL bioreactors containing 250 mL of synthetic must and equipped with fermentation locks to maintain anaerobiosis. Fermentations were monitored by a fermentation robotic device (PhenOFerm) that determines CO_2_ release by hourly weight measurements.

### Chemical and standards

Sulfur compound standards were purchased from Sigma Aldrich (St Louis, MO) EtSH [75-08-1], DMS [75-18-3], DES [352-93-2], thiophene (TP) [110-02-1], SMTA [1534-08-3], DMDS[624-92-0],ETA [625-60-5], DEDS[110-81-6], 3-(methylthio)-propanal (MAL) [3268-49-3], 2ME [60-24-2], MTHTP [13679-85-1], 2MTE [5271-38-5], E3MTP [13327-56-5],3MTPAc [16630-55-0], 3-mercapto-1-propanol (3MP) [19721-22-3 ME [505-10-2], (ETP)[18721-61-4], MTB [20582-85-8], 3MTPA [646-01-5] and ethyl(methylthio) acetate (EMTA) [4455-13-4].

### GC-MS calibration procedure

A synthetic wine was made by dissolving 6 g of L-malic acid in 1 L of a 12% ethanol solution, and the pH was adjusted to 3.3 with 1M NaOH. Standard solutions of 10 g/L were individually prepared in cooled ethanol (−20°C) and stored at −20°C. An internal standard solution was made by dissolving 10 g/L (w/w) of TP and 10 g/L (w/w) EMTA in ethanol and stored at −20°C.

Different volumes of sulfur compounds standard solutions were added to synthetic wine to obtain standard curves by plotting the sulfur response ratio of the target compound and the internal standard against the concentration ratio.

### Light VSCs determination

Lighter volatile sulfur compounds (EtSH, DMS, DES, SMTA, DMDS, ETA and DEDS) samples were prepared adding 7 mL of synthetic medium sample and 25 µL of internal standard (TP) to an empty 15 mL screw cap headspace vial. No further sample preparation was necessary.

Samples were transferred from the sample tray to the Dynamic Headspace System (DHS) module at 30°C. Analytes in the headspace vial were purged after 5 min incubation with 400 mL of nitrogen gas at a 30 mL/min flow rate and trapped at 25°C on a Thermal Desorption Unit (TDU) tube packed with Tenax TA (020810-005-00). The TDU tube was transported and desorbed in the TDU programmed from 20 (held for 0.5 min) to 270°C (held for 3 min) with a rate of 60°C/min. Desorbed compounds were focused at −30°C on a Tenax packed liner in the CIS (012438-010-00) (PTV inlet). After desorption, the inlet was programmed from −30°C at 270°C at 12°C/s (held for 7 min) to inject trapped compounds onto the analytical column. The injection was performed in the PTV Solvent Vent mode with a total flow of 17 mL/min, a septum purge flow of 3 mL/min, a purge flow to split vent of 13 mL/min at 0.1 min and a vent flow of 50 mL/min.

The compounds were separated by the 7890 GC system (Agilent, Santa Clara, CA) coupled to a 5975C single quadrupole mass spectrometry detector (Agilent). The instrument was controlled with Maestro Software Control (Gerstel, Mülheim an der Ruhr, Germany) and the data analyzed with Chemstation Software (Agilent)). The gas chromatograph was fitted with a 30 m × 0.25 mm ZB WAX (Phenomenex, Torrance, CA), 0.1 µm film thickness. The column temperature was programmed from 40°C (held for 0.5 min) to 100°C at 5°C/min, to 150°C at 15°C/min, to 235°C at 7.5°C/min, to 250°C at 15°C/min (held for 1 min). The carrier gas was helium (BIP quality, Air Liquide, Paris, France) at a flow rate of 1 mL/min in constant flow mode, average velocity 36 cm/s. The mass spectrometer quadrupole temperature was set at 150°C, source was set at 230°C and transfer line held at 240°C. For quantification, mass spectra were recorded in selected ion monitoring (SIM) using positive ion electron impact at 70 eV. The ions monitored in SIM runs are shown in Table 1. For each compound, the ion in bold was used for quantification and the other ions were used as qualifiers.

### Heavy VSCs determination

Heavier sulfur compounds (2ME, MTHTP, 2MTE, E3MTP, 3MTPAc, 3MP, ME, ETP, 4MTB and 3MTPA) were extracted using double liquid-liquid extraction (DLLE). A total of 5 mL of each sample were placed in a 15 mL pyrex tube with a Teflon cap, 20 µL of internal standard solution and 1 mL of dichloromethane were then added to the solution. The mixture was shaken during 20 min on a shaker plate at 150 rpm and the samples were then centrifuged at 2000 rpm for 20 min at 4°C. The organic phase was collected with a crystal syringe into a 4 mL vial. This procedure was repeated twice. The organic phase was then dried with 20 mg Na_2_SO_4_ and transferred to a 1.5 mL vial. The sample was evaporated under nitrogen flux to a final volume of 0.5 mL and transferred to an insert vial.

Samples were analyzed with a Trace 1300 GC system gas chromatograph (ThermoFisher Scientific, Waltham, MA) equipped with a Thermo Scientific Triplus RSH Sampler used in liquid mode, and coupled to a Thermo Scientific ISQ 7000 single quadrupole mass spectrometry detector (ThermoFisher Scientific). The instrument was controlled and the data analyzed with the Chromeleon 7.2 software (ThermoFisher Scientific). The gas chromatograph was fitted with a 30 m × 0.25 mm DB-FFAP, 0.25 µm film thickness (Agilent). The carrier gas was helium flow rate 1.0 mL/min in constant flow mode. The initial temperature is held for 0.5 min. Then it increases by 1°C/min to 45°C, by 3°C/min to 120°C, then by 3°C/min to 120°C and finally 20°C/min to 240°C. Final temperature is held during 8 min. The injector was held at 250°C. The sample volume injected was 2 μL in splitless mode. The mass spectrometer quadrupole temperature was set at 150°C, the ion source was set at 230°C and the transfer line was held at 250°C. For quantification, mass spectra were recorded in Selected Ion Monitoring (SIM) mode with positive ion electron impact at 70 eV.

### Statistical analysis

Data analysis was performed with a statistical treatment and graphically (boxplots) represented using the R software version 3.6.1. (http://cran.r-project.org/). The boxplots were designed according the method described by Tukey (1977). The adjacent values are calculated using 1.5 times the interquartile space (the distance between the first and the third quartile). The principal component analysis (PCA) was carried out with the FactoMineR package (Le, Husson and Josse 2008). Normality of residual distributions and homogeneity of variance were studied using standard diagnostic graphics; no violation of the assumptions was detected.

All the experiments were performed in duplicate or triplicate. To assess the consistency and reproducibility of VSCs measurements, additional IR10 fermentations were carried out in five replicates for two experimental conditions and intra-class correlation coefficients were calculated from the global IR10 dataset using a two-way random model provided by the R package irrICC (Quan and Shih 1996). No significant variation was detected in the independent determination of VSCs concentrations in the seven biological replicates. Furthermore, the intra-class correlation coefficients ranged between 0.95 and 1, resulting in a mean intra-measurement reliability of 98%. (Supplementary data 1). This reproducibility analysis indicated the feasibility of our approach for assessing the incidence of environmental variables on VSCs formation.

## RESULTS

### Diversity in VSCs production among *S. cerevisiae* strains

**Figure 1.**
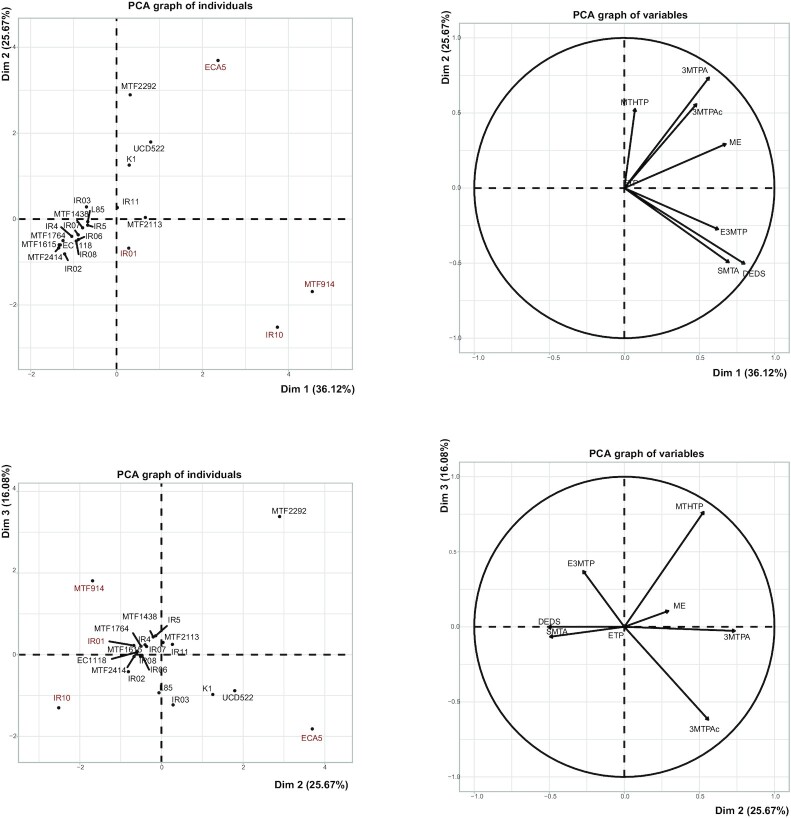
Principal component analysis for volatile sulfur compounds production (MTHTP, ETP, 3MTPA, 3MTPAc, ME, E3MTP, SMTA and DEDS) by 22 *S. cerevisiae* strains. The first three dimensions (Dim1, Dim2 and Dim3) explain respectively 36.73%, 25.21% and 16.08% of the variability in VSCs production between individuals.

To have an overview of the production of VSCs by *S. cerevisiae* wine yeasts, fermentations were carried out using 22 different strains (Table 1) on a chemically-defined grape must containing 200 g/L sugars and 200 mg N/L with a non-limiting pantothenic acid concentration (1 mg/L). The comparison of the production profiles of 17 VSCs measured at the end of fermentation revealed an important variability in the formation of these molecules, depending on their nature (Fig. 1).

First, only 10 (SMTA, ETA, MTHTP, 2MTE, E3MTP, 3MTPAc, ME, ETP, 3MTPA and 2ME) of the 17 compounds analyzed were detected in the wines. The concentrations of the other molecules (EtSH, DMS, DES, DMDS, DEDS, 3MP and 4MTB) were below the detection threshold, indicating either a very limited, or even non-existent production by yeast or considerable losses through evaporation, due to the low boiling points of these molecules (e.g. EtSH with a 35°C boiling point).

Interestingly, some compound such as ME or MTHTP were synthetized by all the strains, even if in variable amounts, between 1.5 and 6.1 mg/L and from 32 to 515 µg/L for ME and MTHTP in the highest and lowest producer, respectively (Table S2, Supporting Information). Conversely, other molecules were synthetized by some strains only. Thus, SMTA was only detected in four media (fermented by strains IR01, IR10, MTF914 and MTF2113), at concentrations varying between 13 and 47 µg/L, while 10 of the 22 strains were able to produce 3MTPAc at concentrations above the detection threshold, from 24 upto 234 µg/L. As a consequence, important variations existed among strains in their capacity to produce VSCs, and a PCA of the data set, for which the first three dimensions explained more than 78% of the variance (Fig. 1), allowed to differentiate low VSCs producers (as EC1118) from high producers (including ECA5, MTF914, IR10, UCD522 and MET2292). Furthermore, it clearly appeared that VSCs profiles differed between high producers: as an example, ECA5 was characterized by an intensive formation of ME, 3MTPA and 3 MTPAc while MTF914 displayed a great ability to produce SMTA and E3MTP. From these observations, four strains (IR01, MTF914, ECA5 and IR10) with strongly contrasting in VSCs profiles were selected for further investigations.

### Impact of nitrogen availability

First, the impact of YAN availability on fermentation performances and VSCs production profile by the four selected trains was assessed during fermentations with variable YAN concentrations: 100, 200 (control), 300 and 400 mg N/L, with 210 g/L sugars and 1.5 mg/L pantothenic acid.

As expected, YAN availability widely affected the fermentation performances of all the strains, with a strong decrease in fermentation rate and a doubled fermentation duration when nitrogen was limiting (MS100 compared to MS300) (data not shown). However, irrespective of the initial YAN content, all the fermentations were completed, with residual sugar concentrations below 0.6 g/L. Regarding the formation of the main compounds deriving from central carbon metabolism (CCM), no major variations according to YAN concentration were found in the production of glycerol, pyruvic and acetic acids. However, strain ECA5 produced almost five times less acetic acid than the other strains.

It is noteworthy that the production of succinic acid substantially changed depending of YAN initial content in the medium, decreasing with the increase in nitrogen availability (Supplementary data 3). ECA5 was least affected with succinic acid production varying from 0.78 g/L in MS100 to 0.51 g/L in MS400, in comparison with strains IR01 or MTF914 for which succinic acid concentrations reached 1.00 g/L in MS100 and 0.40–0.45 g/L in MS400.

Overall, the quantification of the 10 measurable VSCs at the end of fermentation for each modality revealed important differences in their production profiles according to nitrogen availability, which were more or less pronounced depending on the strain used to carry out fermentation. The only exception was 2-MTE produced at a concentration of around 48 µg/L regardless of the initial YAN content.

**Figure 2.**
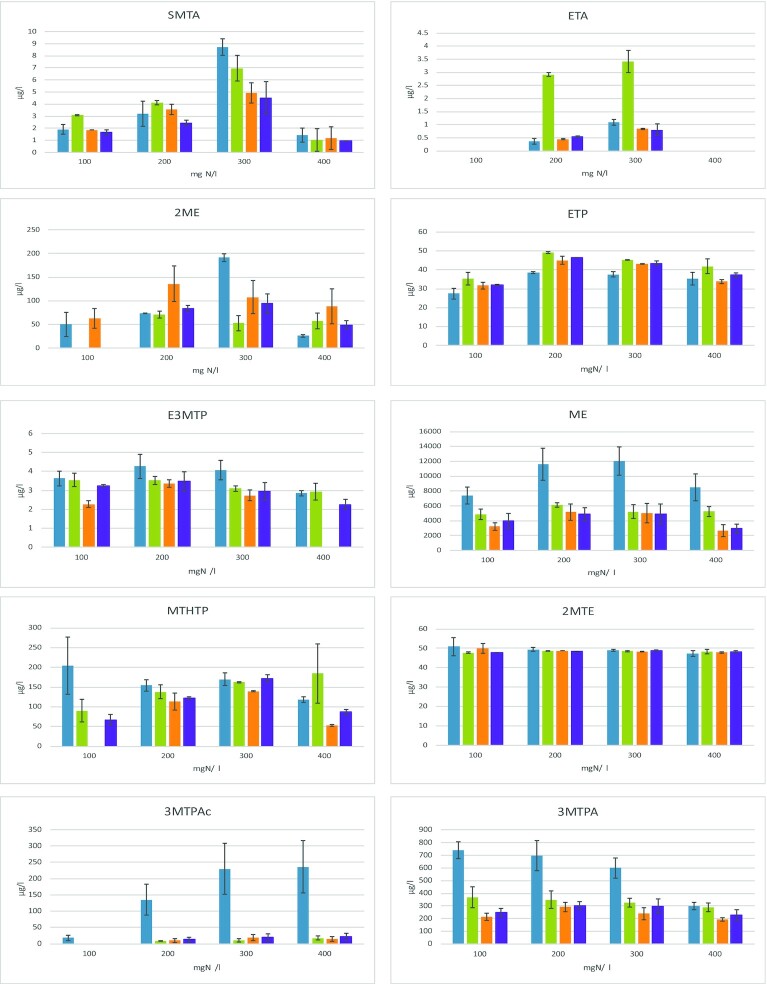
Effect of YAN availability (100, 200, 300 and 400 mg N/L) on the production of VSCs during fermentation on synthetic media. Concentrations of volatile sulfur compounds (SMTA, ETA, 2ME, ETP, E3MTP, ME, MTHTP, 2MTE, 3MTPAc and 3MTPA; µg/L) measured at the end of fermentation by *S. cerevisae* strains ECA5 (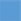), IR01 (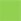), IR10 (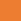) and MTF914 (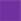). The values represented the averages of duplicate biological experiments ± standard error (SD).

First, the production of SMTA and ETA, both involving the acetylation of either MeSH or EtSH, displayed a bell-shaped curve according to nitrogen availability in the medium, with a maximum production observed at 300 mg/L for all strains (Fig. 2). This maximum value depended on the strain, varying, in the case of SMTA, between 8.7–9.6 µg/L for ECA5 and MTF914 and 6.9–4.9 µg/L for IR01 and IR10. The maximal ETA production was comprised between 0.81 µg/L (MTF914) and 3.4 µg/L (IR01). SMTA production in MS300 was increased 7.5 times (MTF914) and 4.5 times (IR10) with respect to the lowest production (MS400). ETA was not found in MS100 or MS400 for all strains or maybe its concentration was under the limit of detection. Interestingly, 2ME production displayed the same evolution pattern according to YAN availability for all the strains, with a maximum production at around 300 mg N/L.

The VSCs directly deriving from methionine catabolism through the Ehrlich pathway (ME, 3-MTPAc, 3MTPA and E3MTP) were detected in all the wines (Fig. 2). Overall, the production of these molecules exhibited moderate variations depending of YAN, and depending on the strain, the maximum production was obtained for an initial nitrogen content comprised between 200 and 300 mg/L YAN. However, some exceptions to this general pattern were observed. Interestingly, 3MTPAc behaved differently from the other compounds, with a substantial increase with YAN availability. While the final content of 3MTPAc in wine produced by IR01, IR10 and MTF914 in presence of 300–400 mg/L YAN was around 20 µg/L, this compound did not reach the detection limit (5 µg/L) at the end of fermentation carried out with 100 mg/L YAN, except in ECA5 fermentation. In this latter case, the formation of 3MTPAc increased from 18 (100 mg/L YAN) to 236 µg/L (400 mg/L YAN). At the same time, a decrease in the formation of 3MTPA was observed when nitrogen availability in the medium increased, during fermentations achieved with strain ECA5 (from 735 to 301 µg/L) and to a lesser extent with IR01 (from 368 to 288 µg/L). With respect to the 3MTPA production by strains MTF914 and IR10, no significant differences were observed. In addition, ECA5 showed a very high capacity to produce methionine derivatives compared with the other strains. During fermentation with 300 mg N/L, production of ME, 3MTPA and 3MTPAc by ECA5 were 2.3-times, 11-times and 2-times higher than those of the other strains, respectively.

Finally, a slight impact of nitrogen availability was found on the formation of MTHTP and ETP, with variations lower than 30% and a trend towards a lower production at extreme nitrogen levels (100 and 400 mg/L).

### Impact of pantothenic acid concentration

Variable concentrations of pantothenic acid in grape juice, generally varying between 140 and 1380 µg/L) have been reported in literature, depending on variety (Ournac 1970). In synthetic media with a composition mimicking that of natural musts, pantothenic acid was often included at a concentration of 1.5 mg/L (Thomas *et al*. 1993; Guidici and Kunkee 1994; Spiropoulos *et al*. 2000; Ough, Davenport and Joseph 1989). However, these relatively high concentrations do not always correspond to the actual pantothenic acid content found with some varieties or regions, which can be much lower or even limiting (Spayd and Andersen-Bagge 1996; Ribereau-Gayon *et al*. 2000). Thus, *S. cerevisiae* strains MTF914, IR10, ECA5 and IR01 were grown on synthetic must (200 mg N/L and 210 g/L sugars) in presence of variable concentrations of pantothenic acid, i.e. 10, 25, 50, 100 and 250 µg/L, in order to assess the impact of pantothenic acid (from limiting to normal concentrations) on the formation of VSCs.

**Figure 3.**
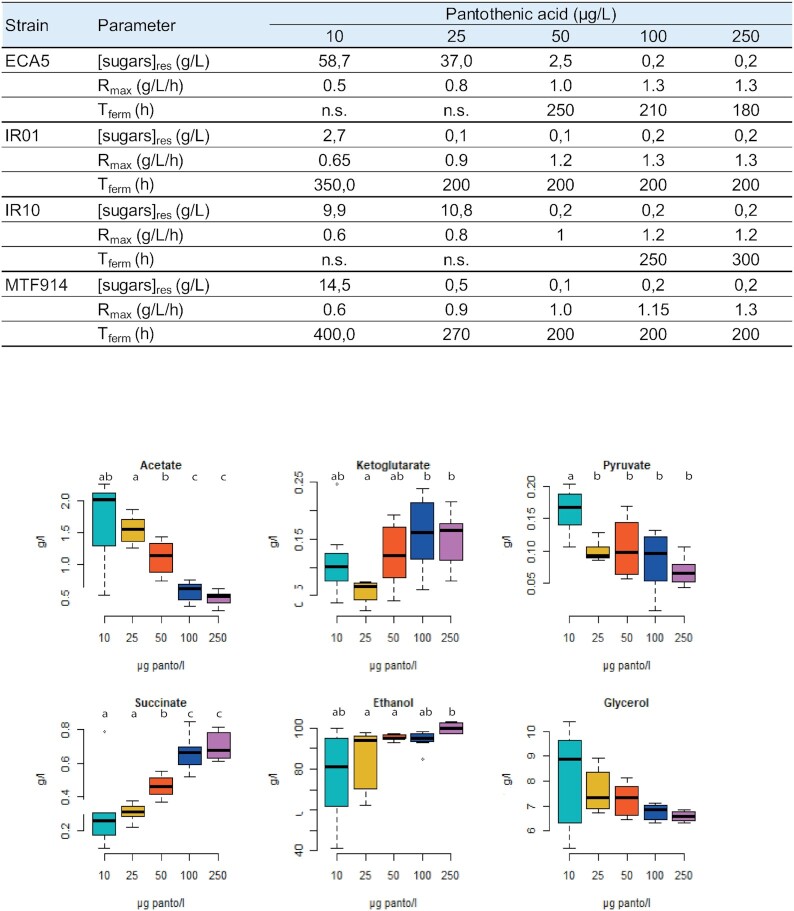
Incidence of pantothenic acid availability on wine fermentation. (A) Fermentation parameters during synthetic media fermentation containing variable concentrations of pantothenic acid, with the strains *S. cerevisiae* ECA5, IR01, IR10 and MTF914. [sugars]_res_: residual sugars concentration; R_max_: maximal fermentation rate; T_ferm_: fermentation duration. n.s.: not significant. (B) Boxplots of the production of central carbon metabolites (acetate, α-ketoglutarate, pyruvate, succinate, ethanol and glycerol; g/L) during fermentation by the *S. cerevisiae* strains ECA5, IR01, IR10 and MTF914 depending on pantothenic acid concentration (10: 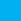, 25 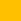, 50 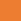, 100 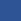 and 250 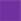 µg/L). Biological duplicates were performed for each condition. The bold central line corresponds to the median. The edges of the boxplots are the 1st and 3rd quartiles. The endpoints of the boxplots are calculated using 1.5 times the interquartile range (the distance between the 1st and 3rd quartile).

The *S. cerevisiae* strains displayed differences in their tolerance to pantothenic acid limitations, as revealed by the comparison of their ethanol production and sugar consumption (Fig. 3). All the strains were able to complete 210 g/L sugar fermentation in presence of 100–250 µg/L pantothenic acid, without incidence on fermentation kinetics. Conversely, providing pantothenic acid at concentrations lower than 50 µg/L resulted in sluggish or even stuck fermentation profiles. ECA5 was the strain most affected by a limitation in pantothenic acid, exhibiting incomplete fermentation in presence of 10 and 25 µg/L pantothenic acid (ethanol production of 43 and 66 g/L, respectively), and a sluggish profile when 50 µg/L vitamin B5 were provided. IR01 was the most tolerant strain to pantothenic acid availability in terms of fermentation duration, except when a strong limitation in this vitamin was imposed (10 µg/L).

Changes in the production of compounds from central carbon metabolism according to pantothenic acid concentration were also observed (Fig. 3). Limited B5 availability resulted in an increase in the production of glycerol (except for ECA5) and acetic acid. Wine glycerol content varied from 6.7–9.7 (10 µg/L pantothenic acid) to 6.3–6.7 g/L (250 µg/L pantothenic acid), and that of acetic acid between 1.3–2.1 (10 µg/L of pantothenic acid) and 0.3–0.6 g/L (250 µg/L of pantothenic acid). Conversely, the production of succinic acid tended to increase with increased pantothenic acid concentration, with concentrations from 0. 1–0.5 g/L (10 µg/L pantothenic acid) to 0.6–0.8 –g/L (250 µg/L pantothenic acid).

**Figure 4.**
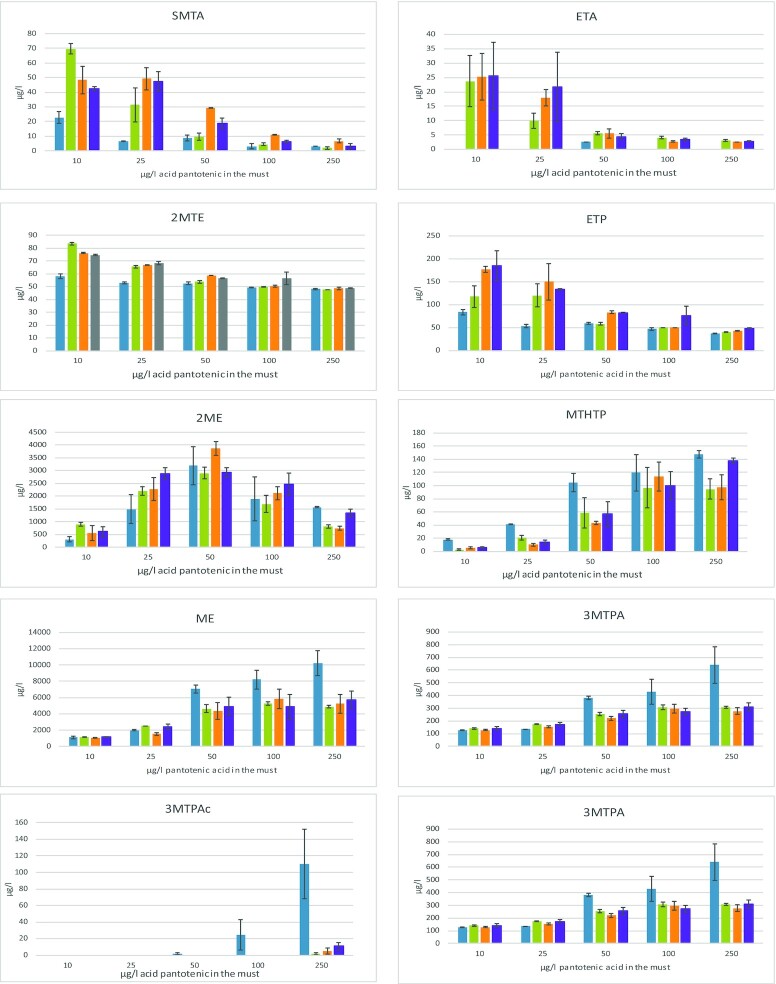
Effect of pantothenic acid availability (10, 25, 50, 100 and 250 µg/L of pantothenic acid) on the production of VSCs during fermentation on synthetic media. Concentrations of volatile sulfur compounds (SMTA, ETA, 2ME, ETP, E3MTP, ME, MTHTP, 2MTE, 3MTPAc and 3MTPA; µg/L) measured at the end of fermentation by *S. cerevisae* strains ECA5 (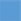), IR01 (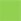), IR10 (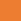) and MTF914 (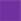). Values represent the average of duplicate biological experiments ± standard error (SD).

A substantial modulation of the production of all VSCs by the availability of acid pantothenic was evidenced (Fig. 4). First, for all strains, the production of thioesters, SMTA and ETA decreased with an increase in pantothenic acid concentration. The only exception concerned ETA formation by ECA5, with production amounts too low to be quantified. Thus, ETA was produced by the strains IR01 IR10 and MTF914 at levels varying from approximately 25 µg/L when pantothenic acid was strongly limiting (10 µg/L) to less than 3 µg/L in presence of 250 µg/L pantothenic acid. Up to a 10-fold decrease in SMTA content in wines was observed between the two extreme pantothenic acid concentrations. Furthermore, the final content of ETP and in a lesser extent of 2MTE displayed the same evolution, being higher under pantothenic acid-limiting conditions.

An opposite impact of the availability of pantothenic acid on the formation of methionine-derivative sulfur compounds was observed, with a strong increase in the formation of ME and 3MTPA when pantothenic acid was provided at concentrations higher than 50 µg/L. The production of ME and 3MTPA by IR01 increased respectively 2-fold and 1.5-fold in limiting and non-limiting conditions. Furthermore, a larger range of variation in the formation of these compounds were found during ECA5 fermentation, with 3.5 and 2.8 times variation factors. As a consequence, any formation of E3MTP and 3MTPAc, that directly originate from 3MTPA and ME, was suppressed during fermentation in presence of 10 and 25 µg/L of pantothenic acid, for all the strains. On the contrary, E3MTP production was approximately 2.1–4.2 µg/L, depending on the strain, for pantothenic acid concentrations from 50 to 250 µg/L. 3MTPAc was only detected when 250 µg/L pantothenic acid was provided during fermentations carried out with strains IR01, IR10 and MTF914. However, during fermentations with ECA5, an increase in 3MTPAc production was observed, from 1.7 (50 µg/L pantothenic acid) to 109 µg/L (250 µg/L pantothenic acid). More broadly, ECA5 appeared as a high producer of compounds deriving from methionine catabolism while wines produced using MTF914 and IR10 showed the highest content in thioesters.

The formation of MTHTP fits the same pattern as methionine derivatives, with concentrations ranging from 2.6–18.1 µg/L during fermentation containing 10 µg/L pantothenic acid to 94.8–147.5 µg/L in presence 250 µg/L pantothenic acid. As a consequence, the formation of MTHTP increased from 8 to 36 times between the lowest and the highest concentrations of pantothenic acid. However, the origin of this molecule remains unknown. Finally, 2ME production exhibited a specific pattern in response to changes in pantothenic acid availability. Increasing vitamin B5 concentration up to 50 µg/L resulted in an increase in 2ME production up to concentrations ranging from 2.9 mg/L (IR01) to 3.9 mg/L (IR10). Over 50 µg/L pantothenic acid, the formation of 2ME decreased for all strains.

### Contribution of methionine and cysteine to VSCs formation

To investigate in which way are produced VSCs and the influence of sulfur amino acids (cysteine, methionine) on their formation, different fermentations were carried out using synthetic media with a modified composition in sulfur amino acids: without either or both sulfur amino acid (0 Cysteine, 0 Methionine, 0 Cysteine Methionine) and with a 20 times increased concentration of either or both sulfur amino acids (20 × Cysteine, 20 × Methionine, 20 × Cysteine Methionine). YAN was adjusted to 200 mg/L for all these experiments

All fermentations reached dryness, even in absence of sulfur amino acid in the initial medium. A slight decrease in fermentation performances without methionine was observed, resulting in 40-hour longer fermentation compared with the other conditions. The modification of the composition of the nitrogen resource in sulfur amino acids did not have any major impact on CCM compounds production. Overall, omitting methionine from the medium resulted in an increased production of succinic acid and, to a lesser extent, of pyruvic acid while the formation of acetic acid was similarly increased, particularly when cysteine was also withheld (Supplementary data 4).

**Figure 5.**
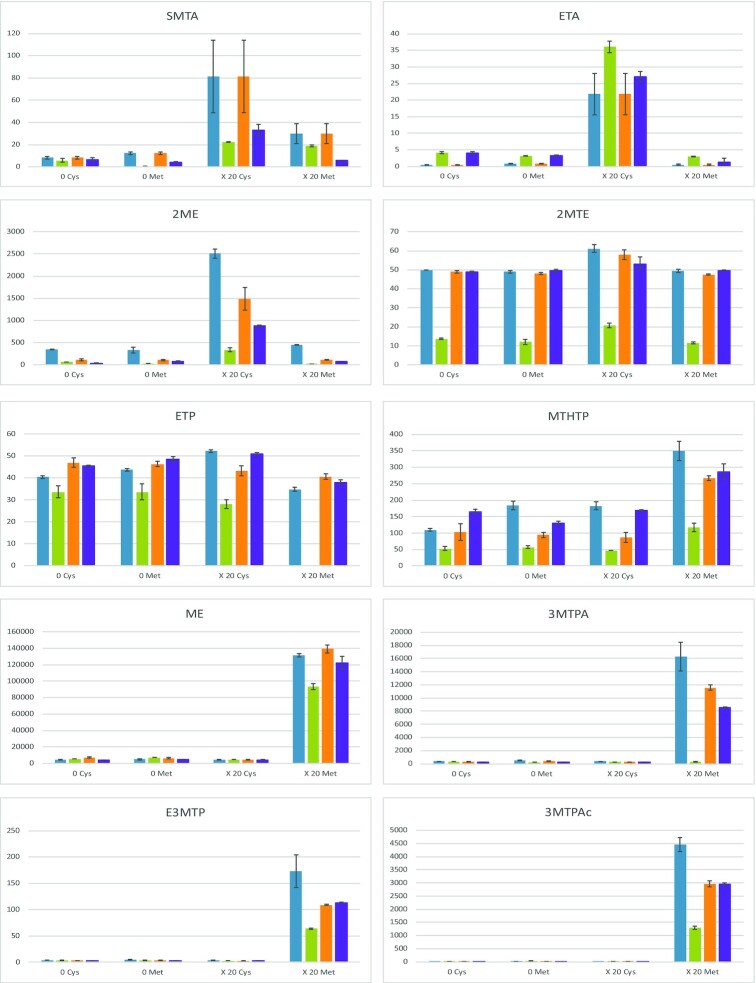
Effect of the availability of sulfur amino acids on the production of VSCs during fermentation on synthetic media. Concentrations of volatile sulfur compounds (SMTA, ETA, 2ME, ETP, E3MTP, ME, MTHTP, 2MTE, 3MTPAc and 3MTPA; µg/L) measured at the end of fermentation by *S. cerevisae* strains ECA5 (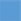), IR01 (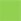), IR10 (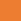) and MTF914 (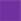). 0 Cys: medium without cysteine; 0 Met: medium without methionine; ×20 Cys: medium with a 20-fold increase in cysteine concentration compared to reference synthetic medium; ×20 Met: medium with a 20-fold increase in methionine concentration compared to reference synthetic medium; YAN concentration was adjusted to 200 mg N/L for each condition. The values represented the averages of duplicate biological experiments ± standard error (SD).

Conversely, the production of all the VSCs showed variations in response to the changes in the availability of methionine and cysteine (Fig. 5). Generally, the formation of a given sulfur-containing molecule was specifically modulated by the concentration of only one of the two sulfur compounds, apart from SMTA that was affected by both methionine and cysteine availability.

Interestingly, the effect of cysteine was focused on four VSCs, SMTA, ETA, 2ME and 2MTE with first a large impact on the production of the two thioesters and 2ME. As an example, the formation of ETA by strains IR10 and MTF914 was nearly suppressed during fermentations without cysteine but was higher than 20 µg/L when this amino acid was provided at 123.2 mg/L in the initial must. Regarding SMTA, increase factors over five were often observed in its production with excess cysteine. Strain IR10 displayed the highest response to cysteine availability, the production of both SMTA and ETA being increased at least 10-fold between conditions without cysteine and with excess cysteine. As an example, the production of SMTA by strain IR01, very low in absence of sulfur amino acids (Fig. 5), was increased to 22.4 µg/L in presence of an excess of cysteine. Finally, we observed that cysteine strongly modulated 2ME production by all the strains. In general, there was great variability between strains in their ability to produce 2ME in absence of cysteine, ranging from a few µg/L (27 µg/L, IR01) to a few mg/L (2.5 mg/L, ECA5) in presence of cysteine. However, excess cysteine resulted in a strong increase in the formation of 2ME regardless the strain, with increase factors of five times (IR01) seven times (ECA5), 13 times (IR10) and 30 times (MTF914).

MTHTP production, that was usually unaffected by environmental conditions such as nitrogen or pantothenic acid amounts, underwent a slight increase as a consequence of the use of a high methionine concentration during fermentation with ECA5, IR10 and MTF914.

As expected, excess methionine resulted in a surplus of methionine derivatives (ME, 3MTPAc, 3MTPA and E3MTP) in all strains. Production of E3MTP, ME and 3MTPA was increased by a factor higher than 30 times between fermentation without methionine and with a methionine excess. At the same time, the formation of 3MTPAc increased 90–230 times (IR01, IR10 and MTF914) up to 370 times (ECA5) between the two conditions. In addition, a 2-fold increase in the production of MTHTP during fermentation was found when methionine was added in excess in the medium compared with conditions without this sulfur amino acid.

The production of SMTA, a sulfur compounds that may be synthetized from intermediates of methionine catabolism, was also affected by methionine availability, but to a lesser extent. The production of this compound was increased by between 1.3 and 2.4 times for IR10, MTF914 and ECA5 but up to 28 times for IR01. SMTA was the sole VSCs showing a response to change in methionine and cysteine availability.

Production of ETP and 2MTE did not change significantly, either with excess methionine or excess cysteine.

## DISCUSSION

In addition to H_2_S whose formation has been extensively investigated (Spiropoulos *et al*. 2000; Linderholm *et al*. 2008; Ugliano *et al*. 2009; Huang *et al*. 2017), a wide range of VSCs can be synthetized during fermentation, with even a low level of production negatively impacting wine quality. In this study, we aimed to provide an overview of the production of these molecules during winemaking and to further explore the poorly studied underlying metabolic bases, the knowledge of which is a prerequisite for the design of strategies to control VSC content in wines.

A total of 17 molecules deriving from sulfur, methionine or cysteine metabolism in living organisms were selected on the basis of their frequency of occurrence in wine. Among these molecules, seven were not detected in any of the wines fermented with 22 different yeasts, including EtSH, sulfides (DMS and DES) and disulfides (DMDS and DEDS). This could be explained by evaporation losses, as some of these molecules (i.e. EtSH and DMS) exhibit very low boiling points (around 35°C), close to fermentation temperature. Another possible explanation is that the fermentation conditions used were not suitable to sustain substantial VSCs formation. In particular, certain grape compounds, not included in the synthetic must, are chemically or biologically converted into VSCs during natural must fermentation. Indeed, previous studies have shown in wines the presence of DMS, DMDS, DMTS and DES, synthetized, at least in part, from S-methyl-L-methionine contained in grape juice (Moreira *et al*. 2002; Segurel *et al*. 2005; Ugliano *et al*. 2009; Deed *et al*. 2019). Finally, interactions between mercaptans and wine components have to be considered to explain the low VSCs production or absence thereof. For example, both a reduction of DEDS to EtSH in presence of free SO_2_ and a strong reactivity of EtSH with metal cations have been reported (Lopez *et al*. 2007; Bobet, Noble and Boulton 1990).

An important variability among *S. cerevisiae* strains in the production of other VSCs included in this study (SMTA, ETA, MTHTP, 2MTE, 2ME, E3MTP, 3MTPAc, ME, ETP and 3MTPA) was showed both qualitatively, in the nature and the profile of synthetized molecules with the formation of 1–6 compounds depending on the strain and quantitatively, in their production amount with increase factors ranging from 1- to 30-fold depending on the compound. These findings complement literature data reporting that the production of light VSCs (DMS, DMDS and DMTS) was highly dependent on the *S. cerevisiae* strains used during fermentation on Trebbiano grape juice (Patrignani *et al*. 2016). More generally, substantial differences were shown in the capacity of yeast species/strains to produce VSCs (Buzzini *et al*. 2005; Tan *et al*. 2012; Seow, Ong and Liu 2010; Moreira *et al*. 2005). Finally, the range of VSCs production detected in this work during *S. cerevisiae* fermentation on chemically defined media was similar to that from fermentations on natural grape juice (Ugliano *et al*. 2009; Moreira *et al*. 2002, 2005), in the direction of formation of these molecules directly related to the intrinsic metabolism of yeasts.

The metabolic pathways described as being involved in VSCs formation (Perpète *et al*. 2006; Landaud, Helinck and Bonnarme 2008) are related to the sulfur assimilation pathway and to the catabolism of the two sulfur-containing amino acids methionine and cysteine. The main inorganic sulfur source for yeast during winemaking is sulphate, found in grape juice in a range of concentrations extending between 150 and 400 mg/L or even higher (Leske *et al*. 1997). Furthermore, adding SO_2_ at contents no higher than 150 mg/L in red wines and 200 mg/L in white wines (EU regulation No. 606/2009), is a common practice in winemaking because this compound primarily acts as a microbiological protection agent, preventing contamination by spoilage microorganisms (yeasts, fungi and bacteria). In addition, this molecule shows anti-oxidant activity, which is essential for the chemical stabilization of harvest compounds. We previously observed that free SO_2_ concentration has a limited impact on VSCs production: among the 17 analyzed VSCs, only a slight increase in the formation of 3MTPAc, 3MPTA and E3MTP was evidenced. Conversely, SO_2_ content strongly influences H_2_S production during *S. cerevisiae* fermentation (Jiranek, Langridge and Henschke 1996). Thus excess sulfur in the cells, which increases with sulfite extracellular content, is likely directly excreted as H_2_S, without triggering changes in the formation of sulfur amino acids and their volatile derivatives.

**Figure 6.**
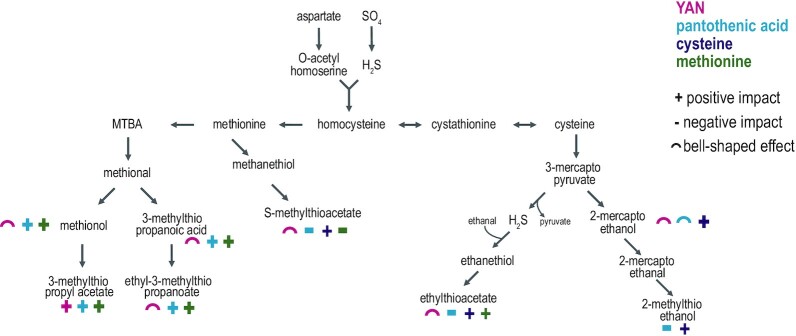
Metabolic pathways involved in methionine and cysteine metabolism. The effect of YAN (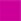), pantothenic acid (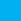), cysteine (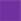)and methionine (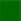) availability on metabolite production are reported as: +: decrease, −: increase, 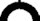: bell-shaped effect.

In this work, the contribution of methionine and cysteine to VSCs production by four *S. cerevisiae* strains was elucidated, either through increasing their concentration or omitting them in the fermentation medium. The role of YAN and pantothenic acid, a vitamin involved in the *de novo* synthesis of sulfur amino acids (Slaughter and McKernan 1988) was also investigated. Overall, the formation of sulfur-containing metabolites was affected by these changes, depending on the metabolic processes involved in their synthesis (Fig. 6).

It has been previously reported that, during natural must fermentation, 3MTPAc, 3MTPA and ME were overproduced in response to methionine supplementation (Moreira *et al*. 2002). In the present study, when provided in excess in a synthetic medium (20-fold increase) during MTF914, IR10, IR01 and ECA5 strains fermentation, at least 65% of initial methionine was recovered as ME, 3MTPAc, 3MTPA and E3MTP. These quantitative data highlight that methionine is the direct precursor of this group of VSCs, through the Ehrlich pathway via α-keto-ϒ-methylthio butyric acid and methional intermediates (Landaud, Helinck and Bonnarme 2008; Perpète *et al*. 2006). Interestingly, the absence of methionine in the medium did not prevent the formation at a basal level of methional derivatives, likely produced from the intracellular pool of *de novo* synthetized methionine to fulfill anabolic requirements. The changes in the production of ME, 3MTPAc, 3MTPA and E3MTP according to the availability of YAN and pantothenic acid support a formation of these molecules mainly through the Ehrlich pathway. First, for the four strains, the production of ME, and to a lesser extent of MTPA and E3MTP reached a maximum level around 200–300 mg/L YAN while the formation of 3MTPAc continuously increased with nitrogen concentration. This behavior is similar to the pattern of variation reported for fusel alcohols, their acetate esters, fusel acids and their ethyl ester derivatives deriving from the metabolism of branched and aromatic amino acids by the Ehrlich pathway (Hazelwood *et al*. 2008; Rollero *et al*. 2015; Dzialo *et al*. 2017). The key metabolic intermediates for the synthesis of these main fermentative aromas are α-ketoacids, shared precursors with the *de novo* synthesis of amino acids. Thus, the modulation of the production of fusel alcohols and acids has been related to the balance between CCM formation of α-ketoacids and their use for amino acids synthesis, both regulated by YAN availability but with different thresholds and magnitude (Rollero *et al*. 2017). Furthermore, nitrogen availability-linked expression of genes *ATF1/2* involved in the acetylation of fusel alcohols in acetate esters, has to be considered to explain the increase in acetate esters together with YAN content (Verstrepen *et al*. 2003; Seguinot *et al*. 2018). Finally, differences between the results presented here and those reported in literature (Moreira *et al*. 2011; Ugliano *et al*. 2009) regarding the impact of YAN on the formation of methional-deriving VSCs can be explained by the use of different ranges of nitrogen concentrations.

Just as ME is formed through the Ehrlich pathway from methionine, it is suggested that 2ME is produced in the same way from cysteine (Dzialo *et al*. 2017). In our experiments, 2ME production behaved as other fusel alcohols deriving from the metabolism of branched chain amino acids (Rollero *et al*. 2015; Dzialo *et al*. 2017), reaching maximum production between 200–300 mg N/L. Also 2ME production was increased more than 10-fold in media with surplus cysteine, suggesting an origin from the Ehrlich pathway with cysteine as precursor. Furthermore, a deficiency in pantothenic acid (under 50 µg/L) drastically inhibited the formation of methional derivatives; this was likely due to a limitation of the capacity of yeast to *de novo* synthetize methionine from carbon central metabolism precursors and H_2_S, because pantothenic acid is essential to the formation of methionine precursors (Sandoval *et al*. 2014). In agreement with this hypothesis, an enhanced formation of H_2_S has been observed as a consequence of pantothenic acid limitation (Wang, Bohlscheid and Edwards 2003; Edwars and Bohlscheid 2007; Bohlscheid *et al*. 2011).

Regarding thioesters, SMTA and ETA are directly synthetized through acetylation of MeSH and EtSH, respectively (Rauhut 2017). EtSH results from the chemical or enzymatic combination of H_2_S with ethanol or acetaldehyde (Rauhut and Kürbel 1994). In addition to a chemical formation from H_2_S (Kinzurik *et al*. 2020), different potential metabolic pathways have been proposed to explain the formation of MeSH during wine fermentation, including demethiolation of methionine or its metabolic precursor α-keto-4-methylthiobutanoic acid (Arfi, Landaud and Bonnarme 2006; Jia *et al*. 2016; Deed *et al*. 2019). Furthermore, chemical interconversions have been established between these highly reactive sulfur-containing molecules (Kinzurik *et al*. 2020). The formation of both SMTA and ETA were increased in response to excess cysteine, confirming the role of H_2_S as precursor of the formation of these thioesters as cysteine catabolism mainly results in H_2_S liberation. Conversely, only the formation of SMTA was modulated by methionine availability. This indicates that a part of excess methionine was converted to MeSH, further acetylated to SMTA, thus invalidating any potential biological or chemical conversion of SMTA into ETA. Interestingly, a pronounced decrease in the formation of thioesters was found when YAN initial concentration was over 400 mg/L. This observation can be explained by a favored use of methionine and cysteine to fulfil anabolic requirements and promote yeast growth in presence of high YAN concentrations; this is achieved at the expense of methionine and cysteine catabolism, and as a consequence, provokes a decrease in MeSH and EtSH formation and of their further acetylation to SMTA and ETA.

A positive correlation between pantothenic acid availability and thioesters production was expected, because this vitamin is the precursor of Acetyl-CoA, both being involved in the formation of the precursors cysteine and methionine and in the acetylation of MeSH and EtSH. Surprisingly, SMTA and ETA production was diminished in presence of high concentrations of pantothenic acid in the medium. It has been reported that the formation of H_2_S was strongly decreased after correcting pantothenic acid deficiency (Edwards and Bohlscheid 2007). Thus, a possible explanation for the impact of pantothenic acid on thioesters formation is a limitation of MeSH and EtSH synthesis related to the decrease of overall H_2_S production abolishing the vitamin limitation.

MTHTP has been measured in reduced wines at concentrations up to 100 µg/L, imparting unpleasant odors to wines (Moreira *et al*. 2005). Little is known on the metabolic pathway responsible for the formation of this compound in yeasts. Both cysteine-conjugated 4-mercapto-4-methylpentan-2-one and methionine have been named as potential precursors of MTHTP (Howell *et al*. 2005; Nawrath *et al*. 2010). The formation of this compound was mostly stimulated by the availability of pantothenic acid and methionine in the medium, supporting the hypothesis of its formation from methionine. Finally, no significant impact of YAN, methionine, cysteine or pantothenic acid availability on the formation of 2MTE and ETP was found.

## Supplementary Material

foab038_Supplemental_File
