# Supplementary material for: How to modulate the formation of negative volatile sulfur compounds during wine fermentation?
Source: FEMS Yeast Res. 2021 Jun 30;21(5):foab038. doi: 10.1093/femsyr/foab038 (PMC8310686; doi:10.1093/femsyr/foab038)

Supplementary data 1: Reproducibility of VSCs determination.

Figure S1: Concentration of VSCs measured at the end of IR10 fermentations on MS200 and MS200 20 X Met. Data from 7 biological replicates.


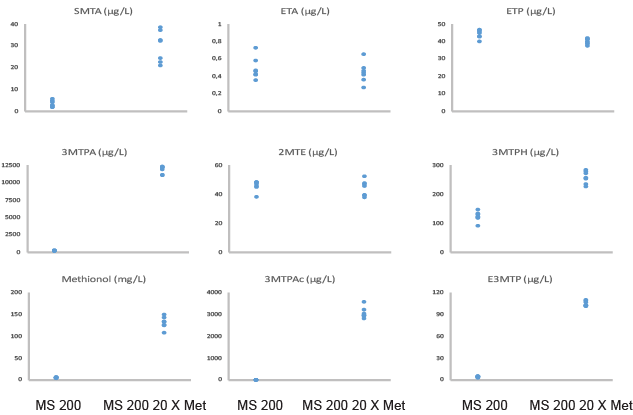


Table S1: Intraclass correlation coefficients (ICC) calculated from VSCs production during IR10 fermentations under 15 different conditions (MS100, MS 200, MS300, MS400, MS200 with cysteine depletion, MS200 with methionine depletion, MS200 with depletion of cysteine and methionine depletion, MS200 with 20-fold extra cysteine, MS200 with 20 –fold extra methionine and MS200 with 20-fold extra methionine and cysteine, MS200 with 10 µg/L pantothenic acid, MS200 with 25, µg/L pantothenic acid, MS200 with 50 µg/L pantothenic acid, MS200 with 100 µg/L pantothenic acid, MS200 with 250 µg/L pantothenic acid). ICCs were calculated from the global IR10 dataset using a two-way random model provided by the R package irrICC (Quan and Shih, 1996); a high ICC (> 0.90) indicates high similarity between values from the same group. S-methylthio acetate (SMTA); 2-methyl-tetrahydrothiophen-3-one (MTHTP); ethyl 3-(methylthio)-propanoic acid(E3MTP); (methylthio)-propyl acetate (3MTPAc); 3-(methylthio)-propanoic acid (3MTPA); ethylthiopropanol (ETP); methionol (ME); 2-(methylthio)-ethanol (2MTE); (ethylthio) acetate (ETA).

| VSC | ICC |
| --- | --- |
| SMTA | 0.99 |
| MTHTP | 0.99 |
| E3MTP | 1.00 |
| 3MTPAc | 0.99 |
| 3MTPA | 1.00 |
| ETP | 0.98 |
| ME | 0.97 |
| 2MTE | 0.95 |
| ETA | 0.95 |

Table S2 : Production of volatile sulfur compounds by 22 *S. cerevisiae* strains. Concentrations in µg/L. S-methylthio acetate (SMTA); diethyl sulfide (DES); 2-methyl-tetrahydrothiophen-3-one (MTHTP); ethyl 3-(methylthio)-propanoic acid(E3MTP); (methylthio)-propyl acetate (3MTPAc); 3-(methylthio)-propanoic acid (3MTPA); ethylthiopropanol (ETP); methionol (ME).

| Strain | SMTA | DEDS | MTHTP | E3MTP | 3MTPAc | 3MTPA | ETP | ME |
| --- | --- | --- | --- | --- | --- | --- | --- | --- |
| IR01 | 25.1 | 0 | 138.0 | 0 | 31.1 | 0 | 0 | 3158 |
| IR02 | 0 | 0 | 0 | 0 | 0 | 0 | 0 | 2079 |
| IR03 | 0 | 0 | 69.1 | 0 | 128.1 | 0 | 0 | 1448 |
| ECA5 | 0 | 0 | 102.8 | 0 | 234.3 | 812.3 | 0 | 5925 |
| UCD522 | 0 | 0 | 77.1 | 0 | 113.1 | 513.2 | 0 | 4244 |
| IR4 | 0 | 0 | 85.8 | 0 | 0 | 0 | 0 | 2511 |
| IR5 | 0 | 0 | 104.2 | 0 | 0 | 0 | 0 | 3750 |
| EC1118 | 0 | 0 | 69.4 | 0 | 0 | 0 | 0 | 1626 |
| IR06 | 0 | 0 | 45.0 | 0 | 0 | 0 | 0 | 3098 |
| IR07 | 0 | 0 | 75.0 | 0 | 0 | 0 | 0 | 3038 |
| IR08 | 0 | 0 | 42.5 | 0 | 0 | 0 | 0 | 2933 |
| L85 | 0 | 0 | 32.3 | 0 | 78.4 | 0 | 0 | 2485 |
| MTF1615 | 0 | 0 | 73.5 | 0 | 0 | 0 | 0 | 1533 |
| MTF1764 | 0 | 0 | 87.3 | 0 | 0 | 0 | 0 | 1815 |
| K1 | 0 | 0 | 45.9 | 0 | 92.5 | 466.6 | 0 | 3179 |
| MTF2414 | 0 | 0 | 55.9 | 0 | 0 | 0 | 0 | 1521 |
| MTF2292 | 0 | 0 | 515.6 | 0 | 0 | 643.2 | 0 | 3272 |
| IR10 | 47.7 | 133.5 | 0 | 0 | 68.7 | 280.9 | 0 | 3352 |
| MTF914 | 19.1 | 96.0 | 105.7 | 16.5 | 37.2 | 233.2 | 0 | 5928 |
| IR11 | 0 | 0 | 89.8 | 0 | 24.1 | 0 | 0 | 5706 |
| MTF2113 | 13.0 | 0 | 110.1 | 0 | 30.0 | 0 | 0 | 6114 |
| MTF1438 | 0 | 0 | 104.1 | 0 | 0 | 0 | 0 | 3347 |

Supplementary data 3: Production of central carbon metabolites during fermentations by the S. cerevisiae strains IR01, ECA5, IR10 and VL1 on synthetic media with different YAN concentrations (MS100: 100 mg N/L; MS200: 200 mg N/L; MS300: 300 mg N/L; MS400: 400 mg N/L).

Table S3: Concentration of sugars (glucose, fructose), alcohols (ethanol, glycerol) and organic acids (pyruvate, succinate, α-ketoglurate and acetate) measured at the end of fermentation by HPLC. Values in g/L.

| Strain | Condition | Acetate | α-ketoglutarate | Pyruvate | Succinate | Ethanol | Fructose | Glucose | Glycerol |
| --- | --- | --- | --- | --- | --- | --- | --- | --- | --- |
| ECA5 | MS100 | 0.301 | 0.105 | 0.164 | 0.803 | 91.8 | 0.4 | 0.0 | 7.07 |
| ECA5 | MS100 | 0.000 | 0.288 | 0.195 | 0.756 | 92.4 | 0.3 | 0.0 | 6.30 |
| ECA5 | MS200 | 0.000 | 0.154 | 0.134 | 0.713 | 96.7 | 0.3 | 0.0 | 6.68 |
| ECA5 | MS200 | 0.141 | 0.132 | 0.058 | 0.667 | 100.9 | 0.4 | 0.0 | 6.81 |
| ECA5 | MS300 | 0.000 | 0.144 | 0.126 | 0.649 | 97.4 | 0.7 | 0.0 | 6.72 |
| ECA5 | MS300 | 0.113 | 0.125 | 0.039 | 0.674 | 102.4 | 0.4 | 0.0 | 6.98 |
| ECA5 | MS400 | 0.000 | 0.081 | 0.125 | 0.518 | 98.6 | 0.7 | 0.0 | 6.59 |
| ECA5 | MS400 | 0.043 | 0.133 | 0.068 | 0.510 | 105.8 | 0.6 | 0.0 | 7.03 |
| IR01 | MS100 | 0.284 | 0.022 | 0.074 | 1.955 | 84.5 | 14.4 | 1.1 | 9.53 |
| IR01 | MS100 | 0.571 | 0.206 | 0.108 | 1.059 | 91.8 | 0.3 | 0.0 | 7.45 |
| IR01 | MS200 | 0.474 | 0.126 | 0.083 | 0.853 | 96.3 | 0.3 | 0.0 | 6.67 |
| IR01 | MS200 | 0.524 | 0.128 | 0.044 | 0.641 | 101.9 | 0.3 | 0.0 | 6.75 |
| IR01 | MS300 | 0.444 | 0.056 | 0.059 | 0.778 | 96.9 | 0.4 | 0.0 | 6.59 |
| IR01 | MS300 | 0.452 | 0.079 | 0.043 | 0.639 | 103.4 | 0.4 | 0.0 | 6.54 |
| IR01 | MS400 | 0.596 | 0.122 | 0.143 | 0.171 | 99.5 | 0.4 | 0.0 | 6.18 |
| IR01 | MS400 | 0.641 | 0.080 | 0.086 | 0.424 | 107.7 | 0.4 | 0.0 | 6.56 |
| IR10 | MS100 | 0.564 | 0.105 | 0.118 | 1.046 | 90.6 | 0.3 | 0.0 | 7.04 |
| IR10 | MS100 | 0.603 | 0.236 | 0.159 | 1.000 | 91.1 | 0.3 | 0.0 | 6.83 |
| IR10 | MS200 | 0.481 | 0.348 | 0.297 | 0.809 | 101.4 | 0.6 | 0.0 | 6.57 |
| IR10 | MS200 | 0.510 | 0.135 | 0.027 | 0.653 | 102.0 | 0.3 | 0.0 | 6.14 |
| IR10 | MS300 | 0.436 | 0.145 | 0.086 | 0.743 | 98.1 | 0.3 | 0.0 | 6.25 |
| IR10 | MS300 | 0.407 | 0.202 | 0.033 | 0.723 | 102.5 | 0.4 | 0.0 | 6.33 |
| IR10 | MS400 | 0.439 | 0.229 | 0.219 | 0.574 | 91.9 | 0.3 | 0.0 | 6.60 |
| IR10 | MS400 | 0.612 | 0.149 | 0.049 | 0.360 | 105.7 | 0.4 | 0.0 | 6.03 |
| VL1 | MS100 | 0.555 | 0.200 | 0.135 | 1.094 | 90.1 | 0.2 | 0.0 | 7.11 |
| VL1 | MS100 | 0.555 | 0.200 | 0.135 | 1.094 | 90.1 | 0.2 | 0.0 | 7.11 |
| VL1 | MS200 | 0.530 | 0.192 | 0.126 | 0.693 | 100.5 | 0.3 | 0.0 | 7.08 |
| VL1 | MS200 | 0.509 | 0.145 | 0.045 | 0.620 | 102.0 | 0.3 | 0.0 | 6.43 |
| VL1 | MS300 | 0.468 | 0.142 | 0.138 | 0.647 | 97.3 | 0.4 | 0.0 | 6.66 |
| VL1 | MS300 | 0.474 | 0.164 | 0.042 | 0.674 | 104.8 | 0.3 | 0.0 | 6.57 |
| VL1 | MS400 | 0.630 | 0.109 | 0.129 | 0.411 | 99.5 | 0.3 | 0.0 | 6.36 |
| VL1 | MS400 | 0.552 | 0.104 | 0.061 | 0.389 | 105.5 | 0.4 | 0.0 | 6.27 |

Figure S3: Box-plots reprensenting the variability of production of CC Metabolites by the 4 strains depending on YAN availability.


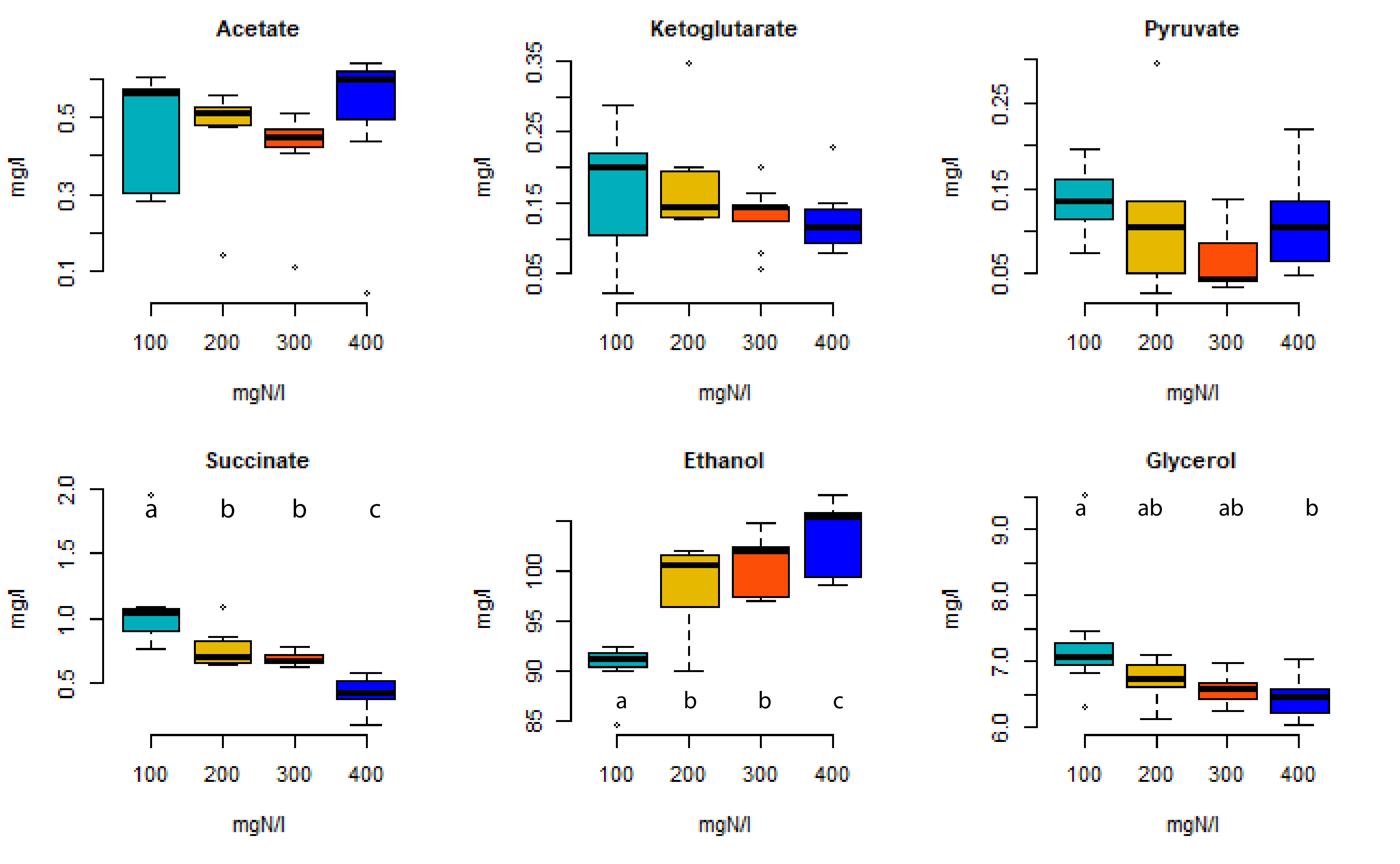


Supplementary data 4: Production of central carbon metabolites during fermentations by the *S. cerevisiae* strains IR01, ECA5, IR10 and VL1 on synthetic media with different amounts of sulfur amino acids (cysteine depletion , methionine depletion , depletion of cysteine and methionine depletion, 20-fold extra cysteine, 20 –fold extra methionine and 20-fold extra methionine and cysteine).

Table S4: Concentration of sugars (glucose, fructose), alcohols (ethanol, glycerol) and organic acids (pyruvate, succinate, α-ketoglurate and acetate) measured at the end of fermentation by HPLC. Values in g/L.

| Strain | Condition | Acetate | α-ketoglutarate | Pyruvate | Succinate | Ethanol | Fructose | Glucose | Glycerol |
| --- | --- | --- | --- | --- | --- | --- | --- | --- | --- |
| IR01 | X20 Met | 0.448 | 0.228 | 0.112 | 0.487 | 96.8 | 0.3 | 0.0 | 6.26 |
| IR01 | X20 Met | 0.476 | 0.149 | 0.074 | 0.593 | 97.7 | 0.3 | 0.0 | 6.12 |
| IR01 | 0 Met | 0.477 | 0.221 | 0.136 | 0.683 | 96.4 | 0.3 | 0.0 | 7.11 |
| IR01 | 0 Met | 0.501 | 0.227 | 0.149 | 0.632 | 97.3 | 0.3 | 0.0 | 7.06 |
| IR01 | X20 Cys | 0.450 | 0.071 | 0.074 | 0.302 | 90.6 | 0.4 | 0.0 | 6.50 |
| IR01 | X20 Cys | 0.446 | 0.186 | 0.101 | 0.289 | 96.3 | 0.4 | 0.0 | 6.39 |
| IR01 | 0 Cys | 0.382 | 0.224 | 0.108 | 0.618 | 96.0 | 0.4 | 0.0 | 6.27 |
| IR01 | 0 Cys | 0.380 | 0.224 | 0.102 | 0.632 | 96.8 | 0.3 | 0.0 | 6.22 |
| IR01 | X20 Cys/Met | 0.465 | 0.179 | 0.067 | 0.517 | 87.3 | 0.3 | 0.0 | 5.55 |
| IR01 | X20 Cys/Met | 0.487 | 0.237 | 0.124 | 0.587 | 98.2 | 0.4 | 0.0 | 6.33 |
| IR01 | 0 Cys/Met | 0.417 | 0.163 | 0.092 | 0.639 | 97.3 | 0.3 | 0.0 | 6.35 |
| IR01 | 0 Cys/Met | 0.395 | 0.237 | 0.170 | 0.683 | 97.4 | 0.4 | 0.0 | 6.51 |
| ECA5 | X20 Met | 0.435 | 0.116 | 0.068 | 0.574 | 95.4 | 0.4 | 0.0 | 5.73 |
| ECA5 | X20 Met | 0.502 | 0.153 | 0.095 | 0.575 | 96.7 | 0.3 | 0.0 | 6.10 |
| ECA5 | 0 Met | 0.466 | 0.173 | 0.145 | 0.792 | 98.3 | 0.3 | 0.0 | 6.81 |
| ECA5 | 0 Met | 0.449 | 0.174 | 0.130 | 0.771 | 98.8 | 0.3 | 0.0 | 6.63 |
| ECA5 | X20 Cys | 0.472 | 0.037 | 0.062 | 0.711 | 96.5 | 0.3 | 0.0 | 6.26 |
| ECA5 | X20 Cys | 0.441 | 0.199 | 0.108 | 0.287 | 96.1 | 0.4 | 0.0 | 6.38 |
| ECA5 | 0 Cys | 0.435 | 0.157 | 0.103 | 0.664 | 96.3 | 0.4 | 0.0 | 6.05 |
| ECA5 | 0 Cys | 0.449 | 0.166 | 0.099 | 0.570 | 97.6 | 0.3 | 0.0 | 6.12 |
| ECA5 | X20 Cys/Met | 0.520 | 0.106 | 0.057 | 0.559 | 85.7 | 0.3 | 0.0 | 6.31 |
| ECA5 | X20 Cys/Met | 0.240 | 0.130 | 0.077 | 0.503 | 97.8 | 0.3 | 0.0 | 6.62 |
| ECA5 | 0 Cys/Met | 0.085 | 0.170 | 0.090 | 0.643 | 97.2 | 0.4 | 0.0 | 6.57 |
| ECA5 | 0 Cys/Met | 0.156 | 0.159 | 0.126 | 0.601 | 85.4 | 0.3 | 0.0 | 6.40 |
| IR10 | X20 Met | 0.487 | 0.224 | 0.097 | 0.517 | 96.6 | 0.3 | 0.0 | 6.95 |
| IR10 | X20 Met | 0.507 | 0.204 | 0.091 | 0.454 | 97.2 | 0.3 | 0.0 | 6.75 |
| IR10 | 0 Met | 0.501 | 0.158 | 0.117 | 0.766 | 97.8 | 0.3 | 0.0 | 7.30 |
| IR10 | 0 Met | 0.378 | 0.248 | 0.122 | 0.806 | 78.4 | 0.3 | 0.0 | 7.47 |
| IR10 | X20 Cys | 0.431 | 0.242 | 0.098 | 0.644 | 93.7 | 0.3 | 0.0 | 6.77 |
| IR10 | X20 Cys | 0.446 | 0.159 | 0.093 | 0.689 | 97.2 | 0.5 | 0.0 | 7.21 |
| IR10 | 0 Cys | 0.477 | 0.147 | 0.089 | 0.616 | 98.3 | 0.4 | 0.0 | 6.52 |
| IR10 | 0 Cys | 0.599 | 0.105 | 0.084 | 0.579 | 97.9 | 0.3 | 0.0 | 6.51 |
| IR10 | X20 Cys/Met | 0.533 | 0.136 | 0.044 | 0.575 | 91.3 | 0.3 | 0.0 | 6.88 |
| IR10 | X20 Cys/Met | 0.549 | 0.093 | 0.066 | 0.509 | 96.0 | 0.3 | 0.0 | 6.69 |
| IR10 | 0 Cys/Met | 0.443 | 0.149 | 0.102 | 0.622 | 102.2 | 0.3 | 0.0 | 6.75 |
| IR10 | 0 Cys/Met | 0.472 | 0.147 | 0.117 | 0.574 | 97.9 | 0.3 | 0.0 | 6.46 |
| VL1 | X20 Met | 0.502 | 0.057 | 0.043 | 0.566 | 96.9 | 0.4 | 0.0 | 6.30 |
| VL1 | X20 Met | 0.479 | 0.143 | 0.063 | 0.563 | 98.0 | 0.4 | 0.0 | 6.36 |
| VL1 | 0 Met | 0.488 | 0.223 | 0.144 | 1.086 | 97.0 | 0.3 | 0.0 | 7.35 |
| VL1 | 0 Met | 0.441 | 0.202 | 0.108 | 0.898 | 95.8 | 0.7 | 0.0 | 7.22 |
| VL1 | X20 Cys | 0.412 | 0.056 | 0.054 | 0.826 | 96.6 | 0.4 | 0.0 | 6.71 |
| VL1 | X20 Cys | 0.400 | 0.190 | 0.086 | 0.785 | 96.8 | 0.3 | 0.0 | 6.55 |
| VL1 | 0 Cys | 0.403 | 0.187 | 0.083 | 0.741 | 97.5 | 0.4 | 0.0 | 6.35 |
| VL1 | 0 Cys | 0.410 | 0.185 | 0.074 | 0.737 | 86.3 | 0.3 | 0.0 | 6.34 |
| VL1 | X20 Cys/Met | 0.513 | 0.101 | 0.042 | 0.644 | 96.9 | 0.4 | 0.0 | 6.44 |
| VL1 | X20 Cys/Met | 0.552 | 0.169 | 0.080 | 0.489 | 96.8 | 0.3 | 0.0 | 6.40 |
| VL1 | 0 Cys/Met | 0.429 | 0.199 | 0.110 | 0.764 | 96.6 | 0.4 | 0.0 | 6.57 |
| VL1 | 0 Cys/Met | 0.425 | 0.209 | 0.127 | 0.736 | 97.2 | 0.3 | 0.0 | 6.51 |

Figure S4: Box-plots representing the variability of production of CC Metabolites by the 4 strains depending on YAN availability.


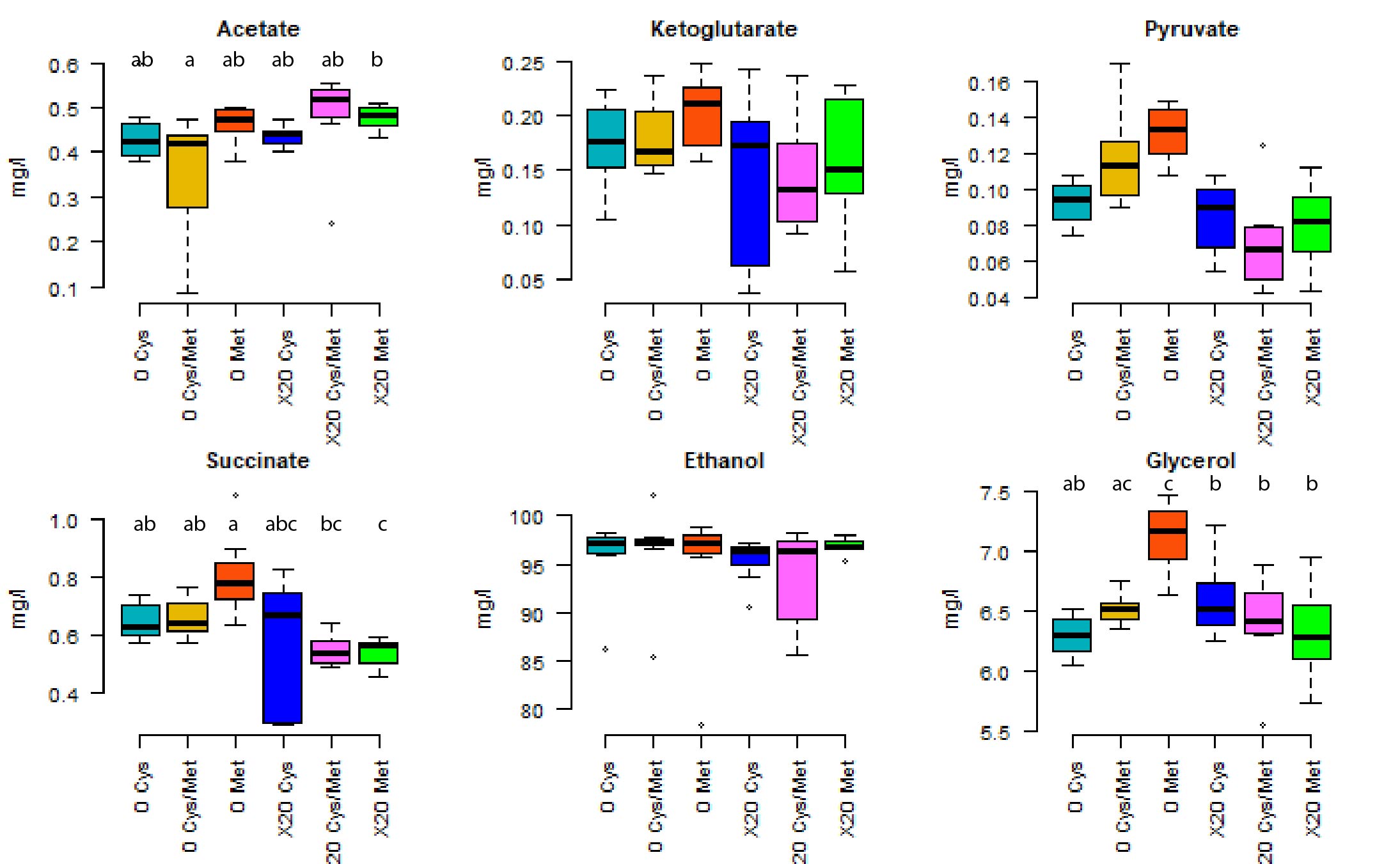

Supplement: foab038_Supplemental_File [file foab038_supplemental_file.docx]
